# Preparing and Analyzing Expressed Sequence Tags (ESTs) Library for the Mammary Tissue of Local Turkish Kivircik Sheep

**DOI:** 10.1155/2017/9604762

**Published:** 2017-01-23

**Authors:** Nehir Ozdemir Ozgenturk, Zehra Omeroglu Ulu, Salih Ulu, Cemal Un, Kemal Ozdem Oztabak, Kemal Altunatmaz

**Affiliations:** ^1^Faculty of Art and Science, Molecular Biology and Genetics, Yıldız Technical University, Istanbul, Turkey; ^2^Department of Biology, Faculty of Art and Science, Ege University, Izmir, Turkey; ^3^Department of Biochemistry, Faculty of Veterinary Medicine, Istanbul University, Istanbul, Turkey; ^4^Department of Surgery, Faculty of Veterinary Medicine, Istanbul University, Istanbul, Turkey

## Abstract

Kivircik sheep is an important local Turkish sheep according to its meat quality and milk productivity. The aim of this study was to analyze gene expression profiles of both prenatal and postnatal stages for the Kivircik sheep. Therefore, two different cDNA libraries, which were taken from the same Kivircik sheep mammary gland tissue at prenatal and postnatal stages, were constructed. Total 3072 colonies which were randomly selected from the two libraries were sequenced for developing a sheep ESTs collection. We used Phred/Phrap computer programs for analysis of the raw EST and readable EST sequences were assembled with the CAP3 software. Putative functions of all unique sequences and statistical analysis were determined by Geneious software. Total 422 ESTs have over 80% similarity to known sequences of other organisms in NCBI classified by Panther database for the Gene Ontology (GO) category. By comparing gene expression profiles, we observed some putative genes that may be relative to reproductive performance or play important roles in milk synthesis and secretion. A total of 2414 ESTs have been deposited to the NCBI GenBank database (GW996847–GW999260). EST data in this study have provided a new source of information to functional genome studies of sheep.

## 1. Introduction

Turkey is an important country in sheep husbandry and there are 33.2 million sheep in the country [1]. Kivircik sheep spread to the Thrace region, Marmara region, and the North Aegean region. They are fed for quality meat, milk, and wool. They are adapted to adverse environmental conditions and resistant parasites. Birth and adult body weight and daily weight gain are 3.7–4 kg, 50–70 kg, and 263 g, respectively. In addition, lactation period is 180 days, lactation milk yield is 83 kg, and wool production yield is 1.5 kg [2].

Colostrum is the first lacteal secretion from the mammary tissue of the mammals after birth [3]. Compared to normal milk, colostrum is clearly richer in contents of immunoglobulin, growth factors, protein, nonprotein nitrogen, fat, ash, minerals, and vitamins [4]. Chemical composition and immunoglobulin level particularly in colostrums exhibits a change within the first 24 h after birth [5–7]. It is only secreted during the first 72 hours of lactation [8] and the excretion is intense at the first 12–36 hours [7]. Sufficient colostrums intake of newborn lambs in the first days of their lives plays essential role in their healthy growth and reaching to ideal market weight [9]. The raisers are desired to breed very productive animal, so colostrums secretion is a crucially important stage for breeding. Because the compounds of colostrums are important, the genes which are related to colostrums secretion have been the center of interest.

A cDNA library containing the information from the mRNA of a particular tissue or organism is an efficient tool for research on gene structure, function, and manipulation [10]. The production of Expressed Sequence Tags (ESTs) begins with the construction of cDNA libraries. The first description of EST was reported from humans in 1991 [11]. ESTs, which are obtained in the results of sequencing of cDNA clones, are very important data for genomics studies [12].

In our study, we used one of the native breeds known as Kivircik sheep in Turkey. First of all, mammary tissues of Kivircik sheep were collected in two stages, before parturition and after parturition (the secretion of colostrums known to be considerably high), and two different cDNA libraries were constructed from those tissues. By using the bioinformatic tools, the ESTs were analyzed. Finally, obtained ESTs were compared with other genes of distinct organisms found in databases and putative functions of the genes were estimated. We aimed in this study to obtain mammary gland of gene expression profile at prenatal and postnatal stages and to compare the genetic components of colostrums secretion according to these two stages in Kivircik sheep.

## 2. Materials and Methods

### 2.1. Tissue Material

Kivircik sheep (*Ovis aries*) in farm of the Faculty of Veterinary Science at University of Istanbul was used in this study. The mammary tissues from the same sheep were taken by biopsy in 6–8 h during high period of the colostrums secretion after parturition and one week before parturition.

### 2.2. cDNA Library Construction, Quality Controls, and Sequencing

Total RNA was isolated from 0,377 gr prenatal stage and 1,316 gr postnatal stage mammary tissues with the RNeasy Kit (Qiagen). mRNA was made pure from total RNA using the Oligotex Spin-Column Protocol (Oligotex mRNA Mini Kit, Qiagen, Valencia, CA). Two different cDNA libraries for pre- and postnatal stage tissue were established with 0,23 *μ*g and 0,75 *μ*g mRNA, respectively. cDNA libraries were constructed with the CloneMiner cDNA Library Construction Kit according to the manufacturer's instructions (Invitrogen, Carlsbad, CA, USA). Double-stranded cDNA was cloned into pDONR222 vector and transformed into* E. coli* strain DH5 (Invitrogen, Carlsbad, CA, USA). Each cDNA library was plated onto LB-kanamycin agar medium and individual grown colonies were picked into 384-well plates with SOB medium and inoculated overnight. After the addition of glycerol (10% v/v), the library was stored at −80°C.

Plasmid DNA was isolated from casually selected 142 clones with alkaline lysis method [13]. Isolated DNA was digested with Bgl1701 and analyzed by 1% agarose gel electrophoresis for identifying insert size.

As a template, randomly selected 3072 clones were used for PCR amplification of the cloned cDNA by M13 universal primers. Automated sequencing was performed on an automated high-throughput pipeline using the ABI 3730 capillary sequencer (PE Applied Biosystems, Foster City, CA) at the Genome Sequencing Center, Washington University in St. Louis (WUSTL).

### 2.3. Sequence Analysis

For analysis of the raw EST data, the low-quality, adapter, and the vector sequences were removed with Phred software [14, 15] (CodonCode Corp., Dedham, MA). The remaining EST sequences were reprocessed by using “cross-match” program which is application of Phrap for the vector sequence trimming [14, 15].

Prenatal and postnatal period EST sequences were assembled separately into contigs with Contig Assembly Program 3 (CAP3) [16, 17]. The default values were used for all the parameters. Results were evaluated by using BEAP program which was developed for editing and representing of alignment [18]. Putative functions of all unique sequences and contigs were designated by gene homology based on BLAST [19]. For Blastn and statistical analysis of all the EST sequences, Geneious software was used [20]. ESTs that showed high similarity in GenBank were classified according to molecular function, biological process, cellular component, protein class, and pathway, respectively, based on Panther classification [21].

## 3. Results

### 3.1. Characterization of cDNA Libraries

**Figure 1:**
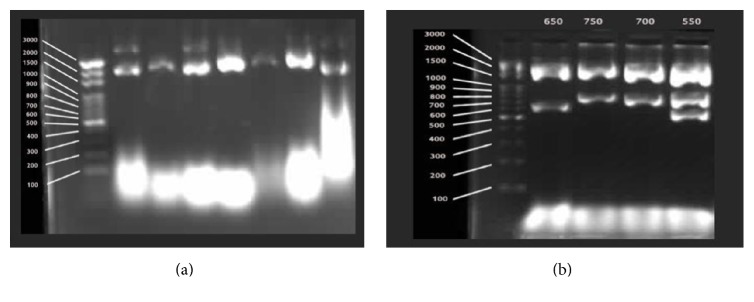
(a) Prenatal cDNA clones obtaining with alkaline lysis method. (b) Cutting of prenatal cDNA clones with Bsp1407I restriction enzyme.

**Figure 2:**
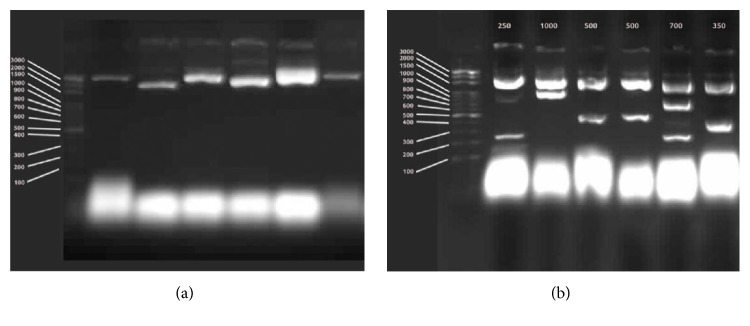
(a) Postnatal cDNA clones obtaining with alkaline lysis method. (b) Cutting of postnatal cDNA clones with Bsp1407I restriction enzyme.

Two different cDNA libraries were constructed at prenatal and postnatal stages from mammary gland tissue. In cDNA library for the prenatal period, there occurred 3.6 × 10^3^ clones and its average insert length was 1 kb (min 200 bp to 1500 bp) (Figure 1). On the other hand, cDNA library for postnatal stage, which consisted of 4.3 × 10^3^ clones, has an average insert length of 0.9 kb ranging from 200 to 1500 kb (Figure 2). After construction of cDNA libraries, 1536 clones were randomly selected and sequenced from prenatal stage library; 1536 clones were randomly selected and sequenced from postnatal stage library. Therefore, 3072 EST sequences were generated.

### 3.2. BLAST Analysis of ESTs

From obtained 3072 EST sequences, raw EST data were processed and base-called with Phred-Cross_match computer program. The EST sequences were trimmed and vector, adapter, and low-quality bases removed. While 318 low-quality sequences were determined in prenatal cDNA library, 340 low-quality sequences were removed in cDNA library which was constructed at postnatal stage (Table 1). The remaining 1218 high-quality prenatal EST sequences and 1196 high-quality postnatal EST sequences were achieved. Putative functions of all unique sequences were designated by gene homology based on BLAST. Blastn analysis was done using Geneious software. Obtained data was evaluated statistically by this program.

**Figure 3:**
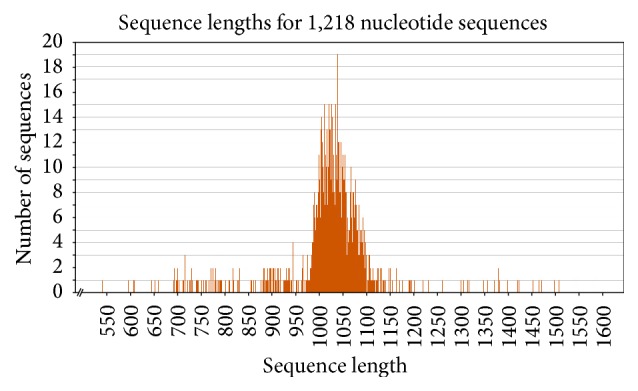
The length of prenatal ESTs.

**Figure 4:**
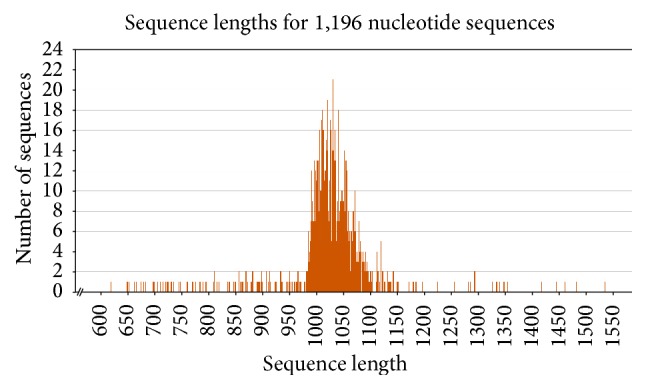
The length of postnatal ESTs.

According to these results, length of prenatal ESTs changes between 542 bp and 1587 bp; the shortest postnatal EST and the longest postnatal EST were 605 bp and 1535 bp, respectively (Table 1 and Figures 3 and 4).

Pursuant to Blastn results of 1218 prenatal ESTs, 154 of them (12.7%) which showed ≥80 similarity or* e* value ≤ 1*e* − 10 to known sequences of other organisms in NCBI were determined. Also the 1001 ESTs (82.3%) showed significant similarities to putative genes with score of below 80% homology and 63 ESTs had no similarity to situated sequences in databases.

Among the 1196 postnatal ESTs, 268 of them (22.4%) showed 80% and higher similarity; 923 ESTs (77.2%) showed similarities with score of <80 bits. Also 38 ESTs did not match any registrated sequences in NCBI.

All 2414 EST sequences were submitted to GenBank. ESTs were registered to NCBI under the accession numbers of GW996847 to GW999260.

### 3.3. GO Analysis of ESTs

**Figure 5:**
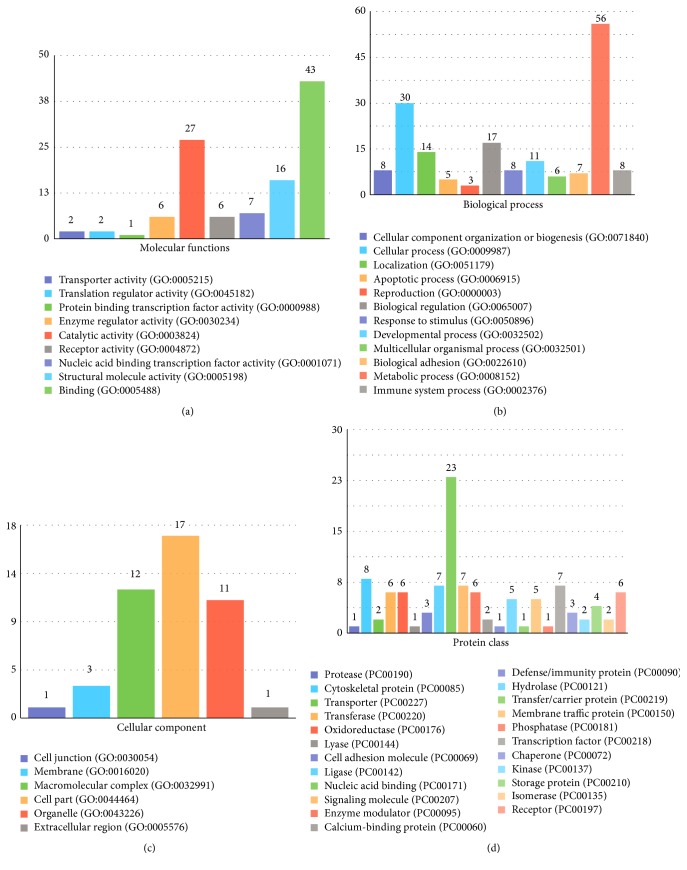
GO terms distribution in the molecular function (a), the biological process (b), the cellular components (c), and the protein class (d) for prenatal ESTs.

**Figure 6:**
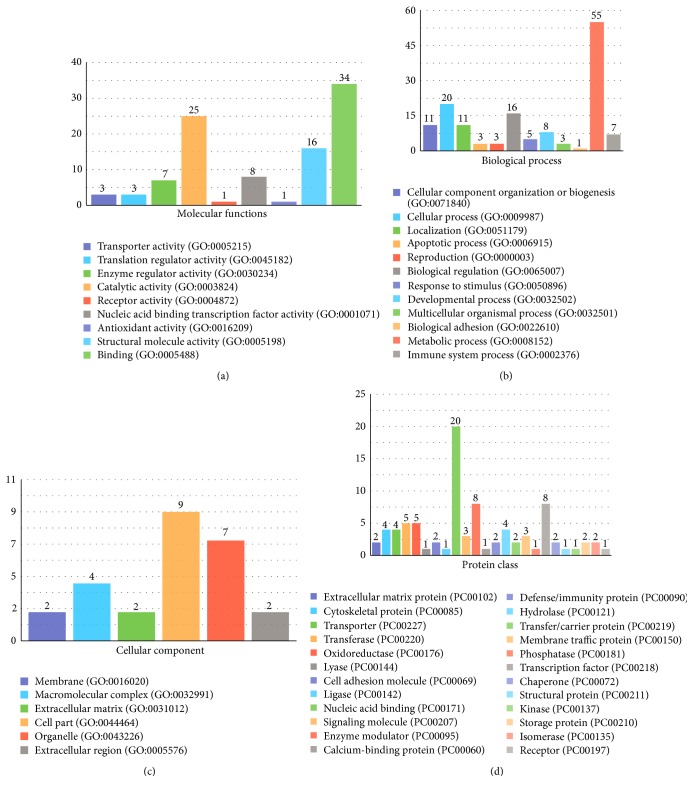
GO terms distribution in the molecular function (a), the biological process (b), the cellular components (c), and the protein class (d) for postnatal ESTs.

Total 422 ESTs which show 80% and over 80% similarity to known sequences of other organisms in NCBI were classified according to GO terms such as molecular function, biological process, cellular components, protein class, and pathway with PANTHER database (Figures 5 and 6). 154 and 268 ESTs of these total ESTs were prenatal and postnatal ESTs, respectively. According to the statistical results of PANTHER of high similarity prenatal and postnatal ESTs, 137 and 127 different putative genes were listed and classified, respectively, by GO terms. 39 prenatal ESTs and 37 postnatal EST were uncategorized.

9 different types of molecular functions were found in prenatal ESTs. The common molecular function GO terms are “binding” which consists of 43 proteins and “catalytic activity” which consists of 27 proteins. Moreover, most of the postnatal ESTs in the molecular function GO term were assigned the same categories, “binding” and “catalytic activity.” In the biological process category that recognizes series of events or molecular functions, 56 and 30 of prenatal ESTs and 55 and 20 of postnatal ESTs were viewed in “metabolic process” and “cellular process,” respectively. The cellular component GO identifies locations at the levels of subcellular structures and macromolecular complexes. In this GO term category, the prenatal ESTs were accumulated more than postnatal ESTs in “cell part,” “macromolecular complex,” and “organelle.” According to protein class GO term classification of Panther database, 23 of prenatal ESTs and 20 of postnatal ESTs most observed “nucleic acid binding” category. On the other hand, Panther database gives information about pathway GO term. When we analyzed prenatal and postnatal ESTs, “B cell activation,” “T cell activation,” and “Huntington disease” categories included some prenatal proteins; moreover, “apoptosis signaling pathway,” “p53 pathway,” and “Parkinson disease” categories contained some postnatal proteins.

### 3.4. Analyzing of Contigs

1218 high-quality prenatal EST sequences and 1196 high-quality postnatal EST sequences were appointed to achieve contig. Fragment assembly was done with the CAP3 software. According to CAP3 result the prenatal stage EST sequences into 23 contigs and postnatal stage EST sequences into 27 contigs were assembled. Furthermore, in prenatal stage contigs, the longest contig is 1394 bp, it is 2068 in postnatal stage contig. The number of singlets is 1164 and 1059, respectively (Table 2).

**Figure 7:**
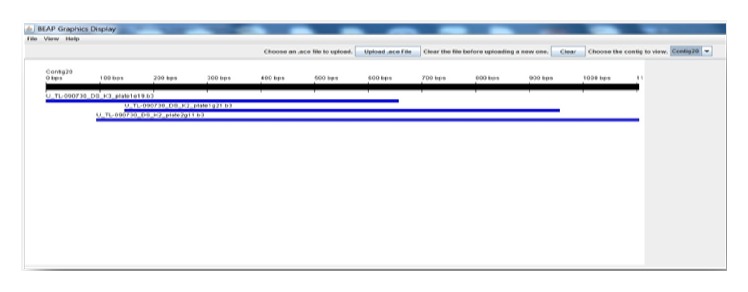
The result of Contig 20 that was obtained by CAP3 with BEAP.

The results which were obtained by CAP3 software were evaluated with the program BEAP which was developed for editing and representing of alignment (Figure 7).

All of the contigs were designated by Blastn in NCBI. The Blastn results of prenatal and postnatal contigs are shown in Tables 3 and 4, respectively.

Among the Blastn results of prenatal contigs, 7 of them showed high similarities. These are a unique protein associated with intracellular transfer of membrane by coated vesicles “clathrin,” protein that plays role in protein synthesis, “elongation factor-1,” a member of the mitochondrial carrier family, “alpha solute carrier family, member 13,” protein that related with immune system, “immunoglobulin gamma one chain,” a gene that encodes the glycodelin protein, “progestagen-associated endometrial protein (PAEP),” a major component of a specific type of lipoprotein called very low-density lipoproteins (VLDLs), and “apolipoprotein E.” Additionally, 10 of 23 prenatal contigs showed similarities to putative different ribosomal proteins in NCBI with score of <80 (Table 3).

As shown in Table 4, 11 of 27 postnatal contigs have similarities with score of ≥80 bits or* e* value ≤ 10^−10^ according to Blastn. 3 postnatal contigs of high similarity postnatal contigs include 3 different ribosomal proteins. In addition, 5 postnatal contigs interacte with calcium and calcium phosphate and self-aggregate to be organized into a supramolecular structure “alpha casein S1 and S2.” The other two contigs show high similarities to putative proteins which are “sperm associated antigen 8 (SPAG8)” and “translation elongation factor 1 alpha.” Important putative genes of the postnatal contigs which showed resemblance below 79% in NCBI are “collagen type III alpha 1,” “nuclear protein, transcriptional regulator,” “beta-2-microglobulin,” “thymosin beta 4,” “beta-casein,” and different ribosomal proteins.

## 4. Discussion

EST projects are powerful tools for analyzing gene expression patterns in a given tissue and/or at a certain stage and the identification of genes [22–24]. These technologies are very important for obtaining genomic sequence information of organisms [25]. In the molecular studies, generation of ESTs is a perfect and unique approach because it allows both expression and estimation and discovery of new genes to be conducted at the same time. Consequently, analysis of expression of a large number of gene profile supplies for scientists to find their functions and facilitates the understanding activity of biological processes in specific tissue or cell of organisms at the certain stage [26].

In this study, we obtained two different EST libraries from Kivircik sheep mammary gland at one week before parturition and 6–8 h after parturition. Because colostrums are only secreted during the first 72 hours after parturition [8], we aimed in this project to achieve putative gene profiling at different time and find the genetic components of colostrums secretion in Kivircik sheep.

The results of the analyzing contig and GO analysis of ESTs and the Blastn analysis of the ESTs showed 80% and higher similarity to putative genes in NCBI. Moreover, we have determined gene expression profile of mammary gland at the different developmental stages, which are prenatal and postnatal.

Table 5 shows Blastn results of some breed-specific expressed genes in all ESTs of prenatal and postnatal ESTs. According to these results, levels of ribosomal protein, transcription factors, and translation factors were found about the same level at both stages. CREB2F, SRF, AKNA, CRTC2, TCF20, and SPDEF transcription factors were observed in prenatal ESTs and also ELF5, NUPR1, BTAF1, TCF12, TFE3, and TCF20 transcription factors were observed in postnatal ESTs. Due to the high level of protein synthesis in the lactation phase, expression of the proteins that participate in ribosomal structure increases at the preparation lactation stage. Also, the genes that have function in transcriptional and translational regulation are expressed highly before parturition [27]. Additionally, we have observed known putative genes related to immune system, growth, and lipid metabolism. These expressed genes which improve quality of milk are “immunoglobulins,” “MHC,” and “beta-2-microglobulin.” Because lactation begins few weeks before parturition [28] and we choose sequenced cDNA clones randomly, these putative genes are found approximately at the same rate in prenatal and postnatal ESTs. Besides, expressions of genes related to milk production are minimal, because the milk is not secreted from mammary gland before the parturition. On the other hand, the milk proteins that affect milk quality are defined as significant milk genes. We have observed their expressions to be high in the lactation stage such as “caseins (alpha-S1, alpha-S2, beta, and kappa casein)” and whey proteins “beta-lactoglobulin” and “alpha-lactalbumin.” Expression of the milk proteins was slightly near the end of gestation, which can be used as a symbol of the maturity of mammary epithelial cells [29].

On the other hand, in respect of GO analysis, GO terms were identified and observed on five categories, such as molecular function, biological process, cellular components, protein class, and pathways. Prenatal and postnatal high-similarity ESTs were classified with Panther database according to GO terms. According to comparison of these results, prenatal and postnatal ESTs were classified almost in the same category.

After our study, using large-scale EST sequencing as strategy, we have constructed Kivircik sheep mammary gland gene expression profiles in different stages and found prelactation and lactation (especially secreting colostrum) specific genes. These results will help us understand the comparison of mammary gland gene expression profiles between two distinct stages and provide new clues for genomic research. Because our ESTs data just only matched the other organisms in NCBI by approximately 50%, it will also provide the increasing number of ESTs of* Ovis* aries in NCBI Genbank database and new genes about* Ovis aries*. This work will improve with the research of different developmental stage of mammary gland.

## Figures and Tables

**Table 1:** The results of analyzing ESTs with Phred software and Geneious software.

	Prenatal period	Postnatal period
EST number	1536	1536
Low quality EST number	318	340
Quality EST number	1218	1196
Average of ESTs (bp)	1023	1026
The shortest EST (bp)	542	605
The longest EST (bp)	1587	1535

**Table 2:** The analysis of prenatal and postnatal contigs by CAP3.

	Prenatal period	Postnatal period
Contig number	23	27
Singlet number	1164	1059
The longest contig (bp)	1394	2068
The shortest contig (bp)	765	994

**Table 3:** The Blastn results of prenatal contigs.

Contig number	Description	Query coverage	Max. ident.	Length of contig (bp)	The number of ESTs in the contig
Contig 1	*Bos taurus* cDNA clone IMAGE: 7944277	56%	92%	768	4
Contig 2	*Bos taurus* ribosomal protein S25, mRNA (cDNA clone MGC: 127761, IMAGE: 7962930), complete cds	45%	92%	1121	2
Contig 3	*Homo sapiens* BAC clone RP11-67M24 from 4, complete sequence	59%	72%	1049	2
Contig 4	*Bos taurus* thymosin beta 4, X-linked, mRNA (cDNA clone MGC: 157367, IMAGE: 8260878), complete cds	73%	94%	860	2
Contig 5	*Equus caballus* clathrin, light chain (Lca) (CLTA), mRNA > dbj|AB495091.1| *Equus caballus* clc mRNA for clathrin light chain, complete cds	85%	96%	1193	2
Contig 6	PREDICTED: *Ovis aries* elongation factor-1 alpha (LOC100125610), mRNA	83%	98%	1083	5
Contig 7	*Ovis aries* ribosomal protein S19 (RPS19), mRNA	47%	98%	1171	4
Contig 8	PREDICTED: *Ovis aries* solute carrier family 25 (aspartate/glutamate carrier), member 13 (SLC25A13), mRNA	80%	96%	1049	2
Contig 9	*O. aries* mRNA for immunoglobulin gamma-1 chain secreted form	88%	87%	1394	2
Contig 10	PREDICTED: *Pantholops hodgsonii* 60S ribosomal protein L32-like (LOC102314866), mRNA	62%	93%	842	2
Contig 11	*Bos taurus* ribosomal protein L31 (RPL31), mRNA > gb|BC102125.1| *Bos taurus* ribosomal protein L31, mRNA (cDNA clone MGC: 127011, IMAGE: 7942112), complete cds	47%	92%	1088	2
Contig 12	*Bos taurus* ribosomal protein L13a (RPL13A), mRNA > gb|BC103039.1| *Bos taurus* ribosomal protein L13a, mRNA (cDNA clone MGC: 128198, IMAGE: 7984430), complete cds	54%	95%	1239	2
Contig 13	*Bos taurus* ribosomal protein S7 (RPS7), mRNA > gb|BC146133.1| *Bos taurus* ribosomal protein S7, mRNA (cDNA clone MGC: 165697, IMAGE: 8068046), complete cds	68%	93%	1036	2
Contig 14	*Capra hircus* serum amyloid A3 (LOC100860781), mRNA	67%	92%	875	4
Contig 15	*Capra hircus* ribosomal protein S18 (RPS18) mRNA, complete cds	73%	95%	765	3
Contig 16	*Bos taurus* progestagen-associated endometrial protein (PAEP), mRNA	84%	92%	1032	4
Contig 17	PREDICTED: *Ovis aries* ribosomal protein S11 (LOC100037666), mRNA	49%	94%	1101	2
Contig 18	*Capra hircus* ribosomal protein S18 (RPS18) mRNA, complete cds	40%	97%	1297	2
Contig 19	*Bos taurus* endothelial differentiation-related factor 1 (EDF1), mRNA > gb|BC102246.1| *Bos taurus* endothelial differentiation-related factor 1, mRNA (cDNA clone MGC: 127033, IMAGE: 7942128), complete cds	74%	88%	1082	2
Contig 20	PREDICTED: *Bubalus bubalis* uncharacterized LOC102396380 (LOC102396380), misc_RNA	64%	83%	890	2
Contig 21	*Bos taurus* ribosomal protein L11 (RPL11), mRNA > gb|BC102524.1| *Bos taurus* ribosomal protein L11, mRNA (cDNA clone MGC: 127967, IMAGE: 7956291), complete cds	63%	95%	965	2
Contig 22	*Bos taurus* ribosomal protein L7a (RPL7A), mRNA	46%	87%	1097	2
Contig 23	*Bos taurus* apolipoprotein E, mRNA (cDNA clone MGC: 127905, IMAGE: 7962554), complete cds	87%	90%	1288	3

**Table 4:** The Blastn results of postnatal contigs.

Contig number	Description	Query coverage	Max. ident.	Length of contig (bp)	The number of ESTs in contig
Contig 1	PREDICTED: *Capra hircus* sperm associated antigen 8 (SPAG8), transcript variant X3, misc_RNA	87%	90%	1015	2
Contig 2	PREDICTED: *Capra hircus* collagen type III alpha 1 (LOC100860938), mRNA	67%	87%	1332	2
Contig 3	PREDICTED: *Capra hircus* nuclear protein, transcriptional regulator, 1 (NUPR1), mRNA	58%	95%	1039	3
Contig 4	*Bos taurus* eukaryotic translation elongation factor 1 alpha 1, mRNA (cDNA clone MGC: 126913, IMAGE: 7928985), complete cds	95%	90%	1992	10
Contig 5	*Bos taurus* ribosomal protein S3 (RPS3), mRNA > gb|BC102090.1| *Bos taurus* ribosomal protein S3, mRNA (cDNA clone MGC: 127206, IMAGE: 7945675), complete cds	82%	92%	1074	2
Contig 6	*Solanum lycopersicum* chromosome ch12, complete genome	30%	67%	2068	2
Contig 7	PREDICTED: *Pantholops hodgsonii* GTPase IMAP family member 5-like (LOC102329836), transcript variant X5, mRNA	17%	91%	1031	2
Contig 8	*C. hircus* mRNA for as1-casein	83%	91%	1072	3
Contig 9	*Bos taurus* beta-2-microglobulin (B2M), mRNA > gb|BC118352.1| *Bos taurus* beta-2-microglobulin, mRNA (cDNA clone MGC: 140690, IMAGE: 8275195), complete cds	79%	89%	1383	2
Contig 10	*Ovis aries* alpha-S1-casein (csn1s1), mRNA > emb|X03237.1| Sheep mRNA for alpha-S1-casein	83%	95%	1283	5
Contig 11	*Ovis aries* alpha-S2-casein (LOC443383), mRNA > emb|X03238.1| Sheep mRNA for alpha-S2-casein	85%	91%	1042	4
Contig 12	*Ovis aries* alpha-S2-casein (LOC443383), mRNA > emb|X03238.1| Sheep mRNA for alpha-S2-casein	82%	95%	1071	13
Contig 13	*Ovis aries* breed Small Tail Han Sheep thymosin beta 4, X-linked (ThymB4X) mRNA, complete cds	60%	95%	1048	2
Contig 14	Sheep mRNA for beta-casein	79%	90%	1487	21
Contig 15	PREDICTED: *Bos taurus* N-ethylmaleimide-sensitive factor, transcript variant 4 (NSF), mRNA	80%	93%	1116	2
Contig 16	*Bos taurus* ribosomal protein S4, X-linked (RPS4X), mRNA > gb|BC102476.1| *Bos taurus* ribosomal protein S4, Y-linked 2, mRNA (cDNA clone MGC: 127573, IMAGE: 7950762), complete cds	84%	92%	1088	2
Contig 17	*Bos taurus* ribosomal protein L27a, mRNA (cDNA clone MGC: 133578, IMAGE: 8058503), complete cds	38%	93%	1361	2
Contig 18	Bovine alpha-S2-casein type A protein (CASAS2) gene, exons 1–18	66%	89%	1054	2
Contig 19	*Ovis aries* alpha-S1-casein (csn1s1), mRNA > emb|X03237.1| Sheep mRNA for alpha-S1-casein	85%	93%	994	2
Contig 20	*Ovis aries* casein kappa (CSN3), mRNA > emb|X51822.1| Sheep kappa-Cn mRNA for kappa-casein	79%	95%	1105	3
Contig 21	PREDICTED: *Capra hircus* 40S ribosomal protein S15-like (LOC102191702), mRNA	45%	96%	1060	2
Contig 22	*Ovis aries* clone 7 ribosomal protein SA (RPSA) pseudogene, complete sequence	50%	97%	1318	2
Contig 23	*Bos taurus* ribosomal protein S3A, mRNA (cDNA clone MGC: 127088, IMAGE: 7943245), complete cds	86%	91%	1069	2
Contig 24	PREDICTED: *Ovis aries* ribosomal protein L6 (RPL6), mRNA	75%	90%	1150	2
Contig 25	PREDICTED: *Pantholops hodgsonii* 40S ribosomal protein S25-like (LOC102318811), mRNA	45%	96%	1079	2
Contig 26	*Bubalus bubalis* alpha-S1-casein mRNA, complete cds	82%	93%	1439	38
Contig 27	*Ovis aries* cell line 1LL2 mitochondrion, complete genome	79%	97%	1059	3

**Table 5:** The percentage and number of some expressed genes in prenatal and postnatal ESTs.

Prenatal ESTs			Postnatal ESTs		
Genes	EST%	Number of ESTs	Genes	EST%	Number of ESTs
Casein (alpha-S1, alpha-S2, beta, kappa)	1,2%	15	Casein (alpha-S1, alpha-S2, beta, kappa)	23,1%	277
Immunoglobulins	2,9%	36	Immunoglobulins	1,4%	17
MHC	1,6%	20	MHC	0,4%	5
Translation factors, elongation factors	2,3%	29	Translation factors, elongation factors	2,5%	31
Transcription factors (CREB2F, SRF, AKNA, CRTC2, TCF20, SPDEF)	0,4%	6	Transcription factors (ELF5, NUPR1, BTAF1, TCF12, TFE3, TCF2)	0,6%	8
Ribosomal proteins	15,1%	184	Ribosomal proteins	14,5%	174
Growth factors	0,3%	4	Growth factors	0,2%	3
Beta-lactoglobulin	2,2%	27	Beta-lactoglobulin	1,9%	23
Lactalbumin	0	0	Lactalbumin	0,6%	8
Beta-2-microglobulin	0,4%	5	Beta-2-microglobulin	0,4%	6
Fatty acid synthase (FASN)	0,5%	7	Fatty acid synthase (FASN)	0,08%	1
Fatty acid binding protein	0	0	Fatty acid binding protein	0,3%	4
